# Editorial: Compensatory growth: an adaptation to environmental stress in plants and animals

**DOI:** 10.3389/fpls.2024.1395038

**Published:** 2024-03-19

**Authors:** Bernard Roitberg, E. Tobias Krause, Chao Li

**Affiliations:** ^1^ Canadian Wood Fibre Centre, Canadian Forest Service, Edmonton, AB, Canada; ^2^ Department of Biological Sciences, Simon Fraser University, Burnaby, BC, Canada; ^3^ Institute of Animal Welfare and Animal Husbandry, Friedrich-Loeffler-Institute, Celle, Germany

**Keywords:** adaptation, compensatory growth, catch-up growth, grassland, forest

Compensatory growth (CG) can be defined as increased growth rate of a previously restricted organism, and has been documented in a wide range of organisms in both the plant and animal kingdoms. Notably, CG can be expressed at the individual, population or even community level as illustrated in the research of this Research Topic. This widely-observed phenomenon has been of interest to scientists for more than a century because it directly impacts our understanding of life-history trade-offs and resource productivity, respectively. Interest in CG continues to be a stable Research Topic of interest with on average 463 paper/year in the last decade (i.e. Web of Science, search term “compensatory growth”, time-period 2014-2023, minimum annual records 398, maximum 522; as of 2024-01-10).

Although compensatory growth is common, it may manifest itself in different ways from exact compensation (often referred to as catching-up growth) to under or over compensation where the comparison is with a non-restricted (i.e. control) group (**Figure 1**). As such, the variability in compensatory growth is as interesting as the phenomenon itself as it often has severe life-history consequences, e.g. costs on other individuals´ traits (Metcalfe and Monaghan, 2001). And, as is the case for many biological phenomena, cross-taxa consideration has the potential to explain both the inner workings and general principles associated with compensatory growth.

**Figure 1:**
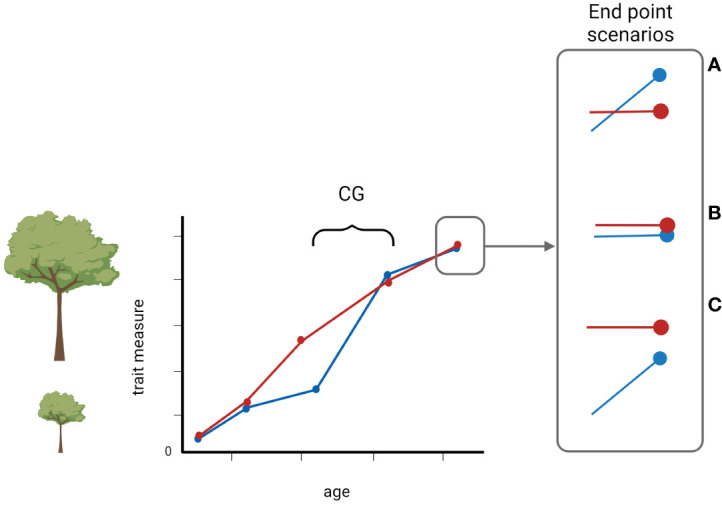
A schematic illustration of compensatory growth over time. The red line denotes normal growth, and the blue line indicates a trait of a measured unit that has experienced an unfavorable condition that resulted in a reduced growth. When the condition improved, the measured unit can display one of the three possible outcomes: **(A)** overcompensation, **(B)** exact compensation, often referred to as catch-up growth, or **(C)** undercompensation. Created with BioRender.com.

In this Research Topic, we have assembled 10 papers on various topics related to compensatory growth.

In Kong et al., the authors used controlled experiments to study the impact of dry-season irrigation and fertilizers on growth of *Eucalyptus* stands via enhanced litterfall decomposition. Their work demonstrates the importance of elucidating the drivers of CG, in this case, release of soil nutrients, a key limiting resource for compensatory growth in trees.

An ecosystem perspective was taken by Zhou et al. to examine how grasslands recover from drought as a form of CG. Furthermore, the authors consider both aboveground and belowground processes among plants and soil-based microorganisms as determinants of ecosystem level response, post-drought. Here, it is also important to keep in mind that CG may be driven by changes in abundance of different species of grassland plants (i.e., ecosystem dynamics) or via increased performance of extant species (i.e., physiological response). There is a further attempt to consider how multiple species respond to stress and post-stress environments by Wang et al.; however, here the focus is on the impact of ammonia-oxidizing bacteria (AOB) on corn (*Zea mays*). In this case, experimentally induced increased presence of AOB, post-drought, led to greater soil nitrification and, as a result, enhanced performance of recipient corn roots and leaves, providing a mechanism to explain compensatory growth in this important crop.

Wu et al. take a mechanistic approach to explaining CG at the forest level. They studied functional traits (i.e., phenotypic traits that interact with the environment) of trees in an old-growth subtropical forest to determine how such trees respond to environmental change. Doing so provides a mechanism for explaining and predicting how forest dynamics are driven by stress (e.g. drought).

Notwithstanding that many interactions between plants and other types of organisms may be neutral or positive (e.g., pollinator-plant mutualisms), insect herbivores can stress their plant hosts by removing plant tissue or vectoring viruses. Moreover, such damage may occur at different plant stages thus adding a ontogenic slant to the problem. Bustos-Segura et al. used an experimental approach to show that lima bean plants are more likely to generate CG or tolerance in the lingo to plant-insect interactions. They found that when such damage is inflicted on seedlings that CG was more likely than when similar damage was applied to juvenile plants mirroring results found in the animal kingdom (e.g., Taborsky, 2006).

Finally, in a series of papers that relate CG to the enhancement of forest productivity since the concept of CG being introduced into forestry research to explain diverse post-thinning stand dynamics by Li et al. (2018), started from establishing a CG conceptual framework for seeking ways of enhancing forest productivity, one of the most desirable outcomes from sustainable forest management, and proposing a research roadmap by Li et al. Accordingly, Li et al. (2021) reviewed modern CG research development and examples from various fields and how different industries benefitted from CG research. Followed by presenting empirical evidence of diverse CG patterns including overcompensation in forest stands across Canada in Li et al. to support the CG conceptual framework. Further, Li et al. and Li et al. (2024) demonstrated that the outcomes of CG are predictable through a statistics-based TreeCG (Tree Compensatory Growth) model. Based on the life-history theory, the TAG (Tree Adaptive Growth) model was developed by Roitberg et al. that proved the previously overlooked overcompensation phenomenon is an expected outcome for trees, in a manner similar to other organisms despite their relatively slow growth and great longevity. As important from an applied-ecology perspective, such responses from individual trees can be scaled up to stands, demonstrating that proper pre-commercial thinning can increase productivity of forests in the long-term.

In sum, this Research Topic provides new insights on how and why CG occurs in nature and, like any good investigation, it raises new questions such as how climate change might alter such responses in the near future. What is clear, however, is that nearly all living organisms are dynamic and will respond in kind as the world changes. CG is an across taxa phenomenon, that although trivial at first glance (growing more when resources become more available after an initial shortage), has complex impact on life histories of organisms.

## Author contributions

BR: Conceptualization, Writing – original draft, Writing – review & editing. EK: Conceptualization, Visualization, Writing – review & editing. CL: Conceptualization, Funding acquisition, Writing – original draft, Writing – review & editing.
